# Primary health care in rural Malawi - a qualitative assessment exploring the relevance of the community-directed interventions approach

**DOI:** 10.1186/1472-6963-12-328

**Published:** 2012-09-20

**Authors:** Peter Makaula, Paul Bloch, Hastings T Banda, Grace Bongololo Mbera, Charles Mangani, Alexandra de Sousa, Edwin Nkhono, Samuel Jemu, Adamson S Muula

**Affiliations:** 1 Research for Health Environment and Development, P.O. Box 345, Mangochi, Malawi; 2 Department of Community Health, College of Medicine, University of Malawi, Private Bag 360, Chichiri, Blantyre 3, Malawi; 3 Centre for Health Research and Development, University of Copenhagen, Thorvaldsens Vej 57, Frederiksberg C, 1871, Denmark; 4 Steno Health Promotion Center, Steno Diabetes Center, Niels Steensens Vej 8, Gentofte, DK-2820, Denmark; 5 Research for Equity and Community Health Trust, P.O. Box 1597, Lilongwe, Malawi; 6 Special Programme for Research and Training in Tropical Diseases, World Health Organisation, 20 Avenue Appia, Geneva 27, CH 1211, Switzerland; 7 Ministry of Health, P.O. Box 30377, Capital City Lilongwe 3, Malawi

## Abstract

**Background:**

Primary Health Care (PHC) is a strategy endorsed for attaining equitable access to basic health care including treatment and prevention of endemic diseases. Thirty four years later, its implementation remains sub-optimal in most Sub-Saharan African countries that access to health interventions is still a major challenge for a large proportion of the rural population. Community-directed treatment with ivermectin (CDTi) and community-directed interventions (CDI) are participatory approaches to strengthen health care at community level. Both approaches are based on values and principles associated with PHC. The CDI approach has successfully been used to improve the delivery of interventions in areas that have previously used CDTi. However, little is known about the added value of community participation in areas without prior experience with CDTi. This study aimed at assessing PHC in two rural Malawian districts without CDTi experience with a view to explore the relevance of the CDI approach. We examined health service providers’ and beneficiaries’ perceptions on existing PHC practices, and their perspectives on official priorities and strategies to strengthen PHC.

**Methods:**

We conducted 27 key informant interviews with health officials and partners at national, district and health centre levels; 32 focus group discussions with community members and in-depth interviews with 32 community members and 32 community leaders. Additionally, official PHC related documents were reviewed.

**Results:**

The findings show that there is a functional PHC system in place in the two study districts, though its implementation is faced with various challenges related to accessibility of services and shortage of resources. Health service providers and consumers shared perceptions on the importance of intensifying community participation to strengthen PHC, particularly within the areas of provision of insecticide treated bed nets, home case management for malaria, management of diarrhoeal diseases, treatment of schistosomiasis and provision of food supplements against malnutrition.

**Conclusion:**

Our study indicates that intensified community participation based on the CDI approach can be considered as a realistic means to increase accessibility of certain vital interventions at community level.

## Background

When the term ‘Primary Health Care’ (PHC) was first conceived in 1974, it was defined as “*a health approach, which integrates at the community level all the elements necessary to make an impact on the health status of the people. Such an approach should be an integral part of the health care system*” [1]. Later, the 1978 Alma Ata Conference redefined PHC as “*essential health care made universally accessible to individuals and families in the community through their full participation and at a cost that the community and the country can afford to maintain at every stage of their development in the spirit of self-reliance and self-determination*” and endorsed PHC as a key strategy for attaining equitable access to basic health care, including treatment and prevention of endemic diseases [2]. Now thirty four years later, PHC implementation remains sub-optimal in Sub-Saharan Africa and access to health interventions is still a major challenge for a large proportion of the rural population. Recently there have been serious attempts to revitalise the PHC concept [3-5]. Yet, health systems in Sub-Saharan Africa continue to be weak and suffer from inadequate mechanisms for delivering PHC services to individuals and communities in need. This maintains the high burden of infectious diseases prevalent in the rural population [6] while hampering effective action against the emerging epidemics of non-communicable diseases. Moreover, health systems lack sustainable frameworks and mechanisms for involving partners from other development sectors and communities at peripheral and operational levels. A well-functioning health system is the backbone to supporting health programmes, and creating healthy populations. Health systems’ strengthening is now recognised as a vital element in the global health agenda to achieve the Millennium Development Goals (MDG) by year 2015.

Many African countries have set up vertical disease-specific control programmes that have since survived, while others have collapsed. Poor conception and exclusion of communities in their initiation have contributed to the failure of these structures to achieve their goals. It has been shown that community participation in health programmes enhances their sustainability and affordability compared to non-participatory programmes, as recurrent costs become more affordable [7,8]. In spite of the mature consensus that community participation is vital to strengthen PHC interventions [9] little progress has been made to involve communities in their planning and implementation. This failure is partially due to the lack of citizen involvement and the social and economic equity necessary to sustain this approach [10].

Malawi has no PHC policy but implements PHC services through the Essential Health Package (EHP) programme. The programme refers to a prioritised but limited package of basic and cost-effective promotive, preventive, curative and rehabilitative health services determined on the basis of scientific and practical experience in service delivery and its ability to have a significant impact on the health status of the majority of the people. The EHP programme was instituted in 2004 following the realisation that PHC as a strategy for achieving *health for all* was not clear, not focused and too general to be attained [11].

Community-directed treatment with ivermectin (CDTi) for onchocerciasis control refers to an approach where the community is given adequate information to become involved in decision-making, organisation, resource mobilisation, selection of community distributors of ivermectin, carrying out or updating community household censuses, distribution of ivermectin, managing and referring side effects and submitting reports of treatment to the nearest health facility [12]. Community-directed intervention (CDI) is defined as a health intervention that is undertaken at the community level under the direction of the community itself [13]. CDI is thus a broad definition of maximum community involvement in public health interventions while CDTi is a narrow definition of maximum community-involvement in the treatment with ivermectin for onchocerciasis control. In CDI the community takes charge of the process of planning and implementing interventions and normally makes use of traditional communication and decision-making procedures in doing so. The role of professional health service providers is to introduce interventions at community level and provide guidance on legal, medical and practical issues. The CDI approach has been used successfully to distribute vitamin A and insecticide treated nets (ITN) by community volunteers as well as in home management of malaria [13,14]. Both CDTi and CDI are rooted in the values and principles of PHC where community participation plays a vital role in the processes of disease control and health development.

A multi-country study conducted in areas that had experienced CDTi demonstrated that CDI can be feasible for integrated delivery of different health interventions in rural Africa [13]. However, limited knowledge is available about its added value for strengthening PHC services in geographical areas with no experience in CDTi. As a basis for the planning and future implementation of PHC interventions using the CDI approach in Malawi, this study examined perceived strengths and weaknesses of existing PHC related strategies and practices, as well as health providers’ and consumers’ perspectives on PHC in two rural Malawian districts without any previous experience on CDTi.

## Methods

### Study area

**Figure 1:**
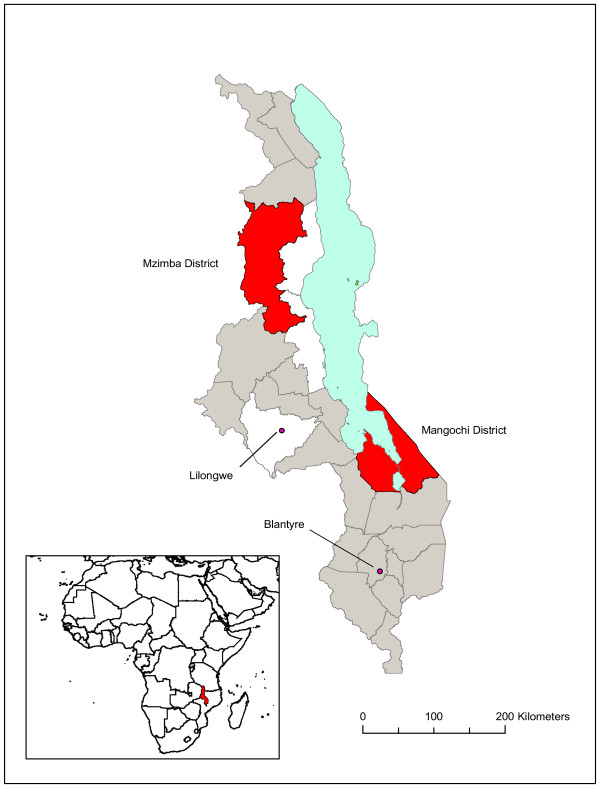
**Map of Malawi showing the two study districts in red.** The dots show the main cities of Lilongwe and Blantyre. The location of Malawi in Africa is shown in the inset.

The study was carried out in 2010 as part of a larger multi-country study involving Cameroon, Kenya, Malawi, Nigeria and Uganda. In Malawi, the study was carried out in two rural districts of Mangochi in the south and Mzimba in the north (Figure 1) where the CDTi strategy for onchocerciasis control has not been applied. Districts were selected based on comparative socio-economic, demographic and health indicators (Table 1). The selection process involved consultations with key Ministry of Health officers, including the Director of Preventive Health Services, the Chief PHC Officer, and District Health Management Team (DHMT) members in the target districts. From an exhaustive list of functioning health centres located in the two target districts, four health centres per district were randomly selected. Subsequently, from the catchment areas of each of the targeted health centres, four villages were randomly selected (Table 2).

**Table 1:** Comparative socio-economic, demographic and health indicators for the two study districts and for Malawi as a whole

**Indicator**	**Mangochi**	**Mzimba**	**Malawi**
Population size/Density^*^	803,602/128	724,873/70	13,066,320/139
DPT3 coverage/All immunizations^**^	82.5%/59.5%	92.1%/72.3%	81.5%/64.4%
Coverage of IPTp (2 doses)^**^	67.2%	88.9%	80.7%
No. of women attending ANC^**^	66.6%	79.7%	70.3%
Maternal mortality rate^***^	400/100,000	210/100,000	984/100,000
Infant mortality rate^**^	104/1,000	80/1,000	76/1,000
Fertility rate^**^	7.2	5.5	6.0
No. of health facilities per 100,000 population^+^	5.1	4.3	4.7
No. of health workers (doctors/nurses/CHW per 100,000 population)^+^	0.8/19.4/71.9	2.0/59.5/73.4	1.9/33.7/76.2
% of population with safe water^***^	70%	74%	67%
Ethnicity^***^	Yao	Ngoni	Various
Predominant religion^*^	Islam (72.2%)	Christian (97.9%)	Christian (82.7%)
Level of illiteracy among women^*^	43.6%	8.2%	54.7%
Major source of income^***^	Fishing	Farming	Farming
% children with fever^**^	36.8%	28.9%	37.9%
% children with fever treated within 24 hours^**^	24.7%	31.0%	28.4%
GDP% of population under the poverty line (1US$)^++^	69.8%	67.5%	65.3%

Sources: ^*^Malawi Census Report, 2008; ^**^Malawi Demographic and Health Survey, 2004; ^***^District Social Economic Profiles for Mangochi and Mzimba, 2003; ^+^A Joint Programme of Works, 2004 &^++^Malawi Poverty Reduction Strategy Paper, 2002.

**Table 2:** Summary of randomly selected health centres and villages involved in the study

**District**	**Names of selected health centres**	**Names of selected villages**
1. Mangochi	1. Nankumba	1. Saiti Tiputipu
		2. Kamangazula
		3. Kansiya
		4. Binali
	2. Phirilongwe	5. Makunula
		6. Nankamwa
		7. Chimwaza
		8. Mtendere
	3. Mase	9. Itimu
		10. Matenganya
		11. Mbalula
		12. Meso
	4. Katuli	13. Kwitunji
		14. Mponda
		15. Sokole
		16. Kasanga
2. Mzimba	5. Kalikumbi	17. Chiza
		18. Kachingwe
		19. Kapinya Zgambo
		20. Border
	6. Mzambazi	21. Vuke
		22. Kaputa
		23. Robert Mtika
		24. Jailosi
	7. Khosolo	25. Chimbomi Mcheleka
		26. Vingistone Kamanga
		27. Eliya
		28. Muduzi Nkhoma
	8. Mbalachanda	29. Kalema Mtambalika
		30. Chagunyuka
		31. Chizapo Kachali
		32. Robert Moyo

### Study population

At national level the Director of Preventive Health Services and the Chief PHC Officer in the Ministry of Health participated in the study. In addition, two representatives from the World Health Organization (WHO) and the United Nations Children’s Fund (UNICEF) participated after being identified by the Director of Preventive Health Services as PHC financial and technical partners. At district level, the study involved selected members of DHMT namely, the District Health Officers, District Nursing Officers and District Environmental Health Officers, Programme Coordinators of PHC, malaria, schistosomiasis and HIV and AIDS. Representatives of some of the development partners of the DHMT, namely Management Sciences for Health (MSH), Catholic Development Commission (Cadecom) and Africare also participated. At health facility level, mostly the in-charges or their representatives who were available on the days of the visit also participated.

At community level all community leaders of the 32 target villages participated in the study. In addition, we purposely selected different groups of respondents from each village, namely men, women, boys and girls, to obtain a diverse community representation and a detailed impression of community perceptions. Ten community members available during the time of the visit were invited to participate in a focus group discussion (FGD). At the end of each FGD session, one participant was randomly selected for a further in-depth interview (IDI).

### Data collection

The study employed qualitative methods to collect data. Key informant interviews (KII) were carried out with the officials at national, district and health centre levels as well as with health services delivery partners at national and district levels. FGDs were carried out with the different homogenous groups of community members such as men, women, boys and girls; one FGD involving ten participants was conducted for each of the groups in each of the target villages. Lastly, in every village IDIs were carried out with a community leader and a randomly selected community member. A summary of the data collected using each of the applied methods is shown in Table 3.

**Table 3:** Summary of type and quantity of data collected at national level and for both target districts combined

**Type of method**	**Unit**	**Number of units**	**Interviews per unit**	**Number of interviews**
KII National	Ministry of Health	1	1	1
KII Partner	National	2	1	2
KII District	District	2	5	10
KII District health worker	District	2	2	4
KII Health Centre	Health centre	8	1	8
KII Village leader	Village	32	1	32
KII Partner	District	2	2	4
FGD	Village	32	1	32
IDI Village member	Village	32	1	32
Total				125

Topics covered during KIIs with health officers and their implementation partners at national and district levels included the nature of PHC elements being implemented at health centre and community levels, priority health issues at community level, health services and their mode of delivery at health centre and community levels, PHC coverage at district level and identification of partner organisations actively involved in PHC. The topics explored using FGD and IDI at health centre and community levels were: perceptions and attitudes of health service providers and consumers towards PHC, role of gender, minority and different socio-political groups, community participation and contribution in health activities, and perceived challenges, opportunities and synergies in PHC delivery.

All tools used at community level were translated to local languages of *Chewa*, *Yao* and *Tumbuka* prior to use. Trained research assistants (4 males and 4 females) conversant with the local languages carried out the data collection. Except during the individual interviews the research assistants worked in pairs where one facilitated the discussion and the other took notes and tape recorded the discussions.

Document reviews were carried at national, district and health centre levels. Some of the documents accessed and reviewed during the study were the Malawi Poverty Reduction Strategy Paper, the Health Sector Wide Approach (SWAp) 2004–2010, Malawi 2004 Demographic and Health Survey, Malawi 2008 Census Report, District Socio-Economic Plans, and Annual Reports. These documents were reviewed in order to get an insight on the national prescription of PHC policy, priority health issues, strategy for delivery of PHC services, effectiveness of the existing PHC interventions, identification of partner organisations involved in PHC, availability of resources for PHC and the challenges, and opportunities and synergies of PHC interventions. Health facility checklists developed during a workshop organised by the multi-country study group in 2008 were used to ascertain the availability of selected vital equipment such as refrigerators, scales, sphygmomanometers and stethoscopes.

### Data analysis

All interviews and FGDs data collected were transcribed and processed using standard word processing software. A computer-assisted qualitative content analysis of the data using Atlas-Ti software was conducted to ensure a standardized and comparable analysis and interpretation of the qualitative data across the study sites. Review of documents made reference to eight key elements of PHC in order to assess the effectiveness and level of implementation of PHC policies and activities. The eight PHC elements are as follows: 1) education concerning prevailing health problems and the methods of preventing and controlling them; 2) promotion of food supply and proper nutrition; 3) adequate supply of safe water and basic sanitation; 4) maternal and child health care, including family planning; 5) immunization against major infectious diseases; 6) prevention and control of locally endemic diseases; 7) appropriate treatment of common diseases and injuries; and 8) provision of essential drugs [2].

### Ethics

Ethical clearance was sought and granted from both the WHO Ethics Review Committee (WHOERC) and the Malawi’s National Health Sciences Research Committee (NHSRC) (NHSRC Approval # 628). Prior to all interviews and discussions oral informed consent was obtained from the participants. The study participants were informed that their involvement in the study was voluntary. The informed consent information sheets administered at community level were translated to local languages.

## Results

### PHC related policies and strategies in Malawi

Document review revealed that Malawi does not have a specific PHC policy. Instead, the government has adopted the EHP as a vehicle for implementing PHC. EHP is supported by a policy document, which defines the minimum package of interventions or services offered to the community, namely: 1) prevention and treatment of vaccine preventable diseases; 2) malaria prevention and treatment, including insecticide treated bed nets (ITN) promotion, intermittent presumptive treatment of malaria during pregnancy (IPTp) and case management; 3) reproductive health interventions, including safe motherhood initiatives, essential obstetric care and prevention of mother to child transmission (PMTCT) of HIV; 4) prevention, control and treatment of tuberculosis and related complications; 5) prevention and treatment of schistosomiasis and related complications; 6) management of acute respiratory infections and related complications; 7) prevention, treatment and care for acute diarrhoeal diseases (including cholera); 8) prevention and management of HIV and AIDS, sexually transmitted infections and related complications including HIV testing and counselling (HTC) and the provision of anti- retroviral therapy (ART); 9) prevention and management of malnutrition, nutrition deficiencies (iodine, vitamin A, iron) and related complications, especially those associated with HIV and AIDS; 10) management of eye, ear and skin infections and related complications; and 11) treatment of common injuries, including emergency care for accidents and trauma and their complications.

### Knowledge about the PHC concept at health facilities within the target districts

The findings showed that there is considerable awareness of the PHC concept at all levels of the health system. The PHC in Malawi is effectively implemented through EHP at both facility and community levels, although at peripheral level, health service providers and consumers were not familiar with the existence of policy documents on PHC or EHP.

*“…the policy… aaaah… to be frank we cannot remember that because it is too long ago when we learnt about the PHC policy… but I think the pillars are the same ten. May be now that you are here you can teach us new things in PHC…”* – KII with a nurse at Mbalachanda Health Centre in Mzimba.

*“…no I am not aware of the policy. Regarding knowledge, I do not think I know much about interventions relating to PHC. I personally think that the policy is needed by clinicians for them to be aware of what is happening other than working blindly without proper guiding principles.”* – KII with a Clinical Officer at Mzambazi Health Centre in Mzimba.

### PHC related services at district level

The study districts have started training volunteers to increase access to health services by the communities. The health centres play a key role in this process and are supported by districts health authorities and their development partners. Districts supply essential drugs and other medical supplies to health centres on a regular basis. In return, health centre staff regularly visits the community to provide services. The beneficiary communities participate in the management of the health centre in collaboration with the health centre personnel through a Health Centre Committee (HCC), which acts as a forum for discussion of health issues. At community level, professional community based health workers referred to as Health Surveillance Assistants (HSA) who are usually employed and salaried by the health system, work closely together with village health committees (VHC) and volunteers to deliver health services to those in need and reports to the health centre. Assessment of services provided at health facility and community levels revealed that some PHC elements receive more attention and resources than others. Advantaged elements included 1) education concerning prevailing health problems, including methods of prevention and control; 2) promotion of food supply and proper nutrition; 3) maternal and child health care, including family planning; 4) immunization; and 5) provision of essential drugs. Disadvantaged elements were 1) adequate supply of safe water and basic sanitation; and 2) appropriate treatment of common diseases and injuries. The lists of advantaged and disadvantaged elements above are prioritised i.e. the first ones mentioned were those that received most attention according to the key informants as exemplified in the following quote for the disadvantaged elements.

*“It* [PHC] *is not very satisfactory because we do not offer comprehensive services, I mean that we do not deliver complete packages, for example HIV/AIDS: we do not offer ART and for TB our patients have to travel all the way to Mzimba to initiate treatment.” –* KII with a Clinical Officer at Mzambazi Health Centre in Mzimba.

We observed an inequitable geographical distribution of PHC service provision, even within the same district, largely as a result of inadequate availability of resources. Some areas were better served than others due to the presence of partner organisations with adequate resources and particular priorities.

“On average 70 percent of people in the district are benefitting from the activities because many areas are still uncovered. What I can say is that PHC is at community level but short of government staff and this is making people benefiting in bits and pieces from the activities.”

*“There is also a problem of improper staff deployment, many HSA were employed but they operate from the Boma* [town] *or health centres instead of operating from the communities.” -* KII with PHC Coordinator Mzimba

*“There are good community health workers but it can be better if they can deploy more health workers so that they can assist us in times of epidemics like cholera and measles.” -* Respondent during an FGD session with girls at Kansiya village in Mangochi.

### Priority health issues within the study districts

While health authorities’ views on priority health issues correspond to those outlined in the EHP policy document, there were marked variations regarding the priority health issues and interventions between the two districts among both the providers and the beneficiary communities (Table 4). An example is whilst the providers in Mangochi prioritised malaria and diarrhoeal diseases the opposite is true for Mzimba where diarrhoeal diseases and tuberculosis were prioritised. Some differences between perceptions of health providers and beneficiaries regarding priority health interventions were also observed, though surprisingly, no major discrepancies were found between women and men nor between groups of adults and youths on their priority health issues (Table 5). While both health providers and consumers shared desires to see interventions against malaria, diarrhoea, malnutrition, measles, HIV and AIDS prioritised, the communities went further by requesting for more services such as transport for referral of patients, drug and vaccine stock outs, water and sanitation improvement and installation of electricity at health facilities. 

**Table 4:** Ranked priority health issues and interventions according to health providers and consumers for both target districts combined

**Priority health issues according to providers**	**Priority health issues according to community**
1. Malaria	1. Measles
2. Diarrhoeal diseases	2. Malaria
3. Pneumonia	3. Lack of safe water
4. Tuberculosis	4. Malnutrition
5. HIV/AIDS	5. Transport
6. Measles	6. Long distances to health centres
7. Malnutrition	7. Vaccine stock outs
8. Schistosomiasis	8. Lack of family planning services
9. Accidents	9. Poor sanitation
	10. Poverty
	11. Lack of electricity
	12. Unwillingness of community health workers to work outside working hours
	13. Insufficient health care personnel
	14. Lower quality of hospital care
	15. Lack of access to free ITN
	16. Absence of NGOs
**Priority health interventions according to providers**	**Priority health interventions according to community**
1. Prevention and treatment of vaccine preventable diseases.	1. Provision of ITN to under five children and antenatal care to women
2. Malaria prevention and treatment – ITN promotion, IPT and case management.	2. Home case management of malaria
3. Reproductive health interventions – including safe motherhood initiatives, essential obstetric care and PMTCT.	3. HTC and PMTCT
4. Prevention, control and treatment of tuberculosis and related complications.	4. Provision of food supplements to malnourished children and mothers
5. Prevention and treatment of schistosomiasis and related complications.	
6. Management of acute respiratory infections and related complications.	
7. Prevention, treatment and care for acute diarrhoeal diseases including cholera.	
8. Prevention and management of HIV/AIDS, sexually transmitted Infections and related complications including HTC and the provision of ARVT.	
9. Prevention and management of malnutrition, nutrition deficiencies- (iodine, vitamin A, iron) and related complications, especially those associated with HIV/AIDS.	
10. Management of eye, ear and skin infections and related complications.	
11. Treatment of common injuries – including emergency care for accidents and trauma and their complications.	

**Table 5:** Number of times (presented in brackets) priority health issues were mentioned during FGD sessions according to age and gender of participants

**Participants**	**Male**	**Female**
Youths (aged 13–18 years)	Malaria (8)	Water (6)
	Cholera (6)	Health centre far (4)
	Water (6)	Diarhoeal diseases (2)
	Malnutrition (4)	Lack of community health worker (2)
	Schistosomiasis (3)	Lack of latrines (2)
	Health centre far (2)	Malaria (2)
	Measles (2)	Malnutrition (2)
	No mosquito nets (2)	Measles (2)
Adults (aged above 18 years)	Health centre far (4)	Malaria (8)
	Measles (4)	Measles (6)
	Water (4)	Water (6)
	Malnutrition (3)	Schistosomiasis (4)
	Cholera (2)	Diarrhoeal diseases (4)
	Malaria (2)	Health centre far (4)
	No community health worker (2)	Poor roads and bridges (4)
	No health worker at Health centre (2)	Health worker unwilling to work during odd hours (2)
	No mosquito nets (2)	Ill-treatment of patients by health worker (2)
		Lack of food (2)

### Health partners engaged in PHC in the study districts

The most common problems cited by both consumers and providers regarding the implementation of PHC were the inadequacy of supplies and shortage of personnel. This contributed to the low quality and quantity of PHC services provided. The 2002 Malawi Poverty Reduction Strategy (MPRS) paper provides a rallying point for development and health partners to participate in development endeavours [14]. The presence of partners such as Icelandic International Development Agency (ICEIDA) and Catholic Development Commission (Cadecom) in Mangochi, and World Vision International (WVI) and UNICEF in Mzimba has helped alleviate the negative impact of the system’s deficiencies. However, in the study districts, the presence of partner organisations at facility and community levels was scarce, not evenly distributed across the districts, and often limited to specific and narrow priority services preventing them from engaging in the delivery of the complete package of PHC services (Table 6). It has been observed that out of 13 partner organisations mentioned as available in Mangochi, only 7 are actively involved in delivery of health services in the district; while in Mzimba, out of the 9 partner organisations mentioned only 4 are involved in health services delivery. Those that are involved in delivery of health services have specific priority health issues and specific geographical areas they are serving within the districts.

**Table 6:** List of development and health partners in the two study districts

**Name of partner/stakeholder**		**Areas of specialty**
	A. Mangochi	
1. Icelandic International Development Agency (ICEIDA)		- Developmental, health and education
2. Catholic Development Commission (Cadecom)		- Developmental, food security
3. Food and Agricultural Organization (FAO)		- Food security
4. Malawi Social Action Fund (MASAF)		- Infrastructure development
5. Save Orphans Ministry (SOM)		- Home based care of orphans
6. Safe Motherhood Project		- Community based reproductive health
7. Namwera Aids Coordinating Committee (NACC)		- HIV/AIDS in community
8. Emmanuel International		- Food security and health
9. Christian Hospitals Association in Malawi (CHAM)		- Provision of curative health services
10. Muslim Association of Malawi		- Health and education services
11. Save the Children		- Education and health in community
12. Population Services International (PSI)		- Social marketing
13. Management Sciences for Health (MSH)		- Essential health services
	B. Mzimba	
1. World Vision International (WVI)		- Food security and health
2. United Nations Children’s and Emergency Fund (UNICEF)		- Health, education, water and sanitation
3. Action Aid		- Development and food security
4. Plan Malawi		- Education and food security
5. Cord Aid		- Health
6. Every Child		- Child protection
7. Africare		- Community development
8. Tovwirane		- HIV/AIDS in community
9. Catholic Development Commission (Cadecom)		- Developmental, food security

### Human, technical and infrastructural resources at health facility level

Study districts were not spared by the national shortage of health personnel. Health centre staff and community health workers felt incapable of meeting the performance targets set by the Ministry of Health. In most health facilities visited (three in Mangochi and two in Mzimba) the infrastructure quality was poor as evidenced by a general lack of maintenance of the buildings and premises; in addition there was inadequate space for in-patients, storage of consumables and equipment. Poor or unavailable transport and communication was observed at all the visited facilities and this hindered effective delivery of PHC. Equipment and supplies in health facilities were mostly erratic and insufficient in all the facilities. Health centres reported lack of feedback from districts regarding their portion of the budget as most of the decisions were made at district level. Such poor involvement of personnel at health centre level in the budgeting and appropriation of resources was considered an impediment towards their sense of programme ownership. Government commitment has been demonstrated by allocation of resources for implementation of EHP (in Malawi, 9.8% of GDP is spent on health of which 11% is spent on EHP [15] and by the implementation of decentralization policy enacted in the 1999 Local Government Act [11]. Yet, a poor macroeconomic environment and the negative impact of the HIV and AIDS epidemic remain major threats to the government capacity to increase per capita health expenditures and to the country’s economic development, respectively.

### Community and health worker perceptions on the quality of PHC related services

Health providers and their partner organisations at district and facility levels perceived that PHC was not being delivered in a comprehensive manner. Community leaders and members viewed the public system as incomplete in covering PHC in all geographical areas and perceived that the partner organisations were the ones filling the gaps left by the formal health system. Communities viewed health workers with suspicion and mistrust, especially when facilities were constantly running out of essential drugs. Health workers reported that because the government did not adequately remunerate them they had to rely on other financially rewarding activities and per diems from attending seminars and workshops. They also thought that communities were mainly attracted to free health services and medical supplies such as ITN. Providers, including partner organisations and communities shared views that PHC should be strengthened through community participation in the planning and implementation of interventions.

*“We have enormous challenges: Human resource; you see that health care workers refuse postings to peripheral health centres. As such we resolve to send them to work on relief duties and such a strategy drains a lot of resources. Just for example last month we spent a third of our budget on relief duties.” -* KII with District Health Manager, Mzimba.

*“There is a village health committee which is looking at the sanitation issue and report to the Health Surveillance Assistant, advise each other if any problem we have to rush to the health centre, we have village fund which helps the sick and support HIV and AIDS patients. We are also digging channels for the piped water (Kalingondi) and each village is contributing US$30 and US$6 contribution for the watchman who is guarding the source and constructing pit latrines.” -* KII with a village head, Mzimba.

### Voluntary groups engaged in PHC services at community level

There were several socio-political groups actively involved in the delivery of various PHC activities within their communities. The most important ones are village health committees (VHC) dealing with health issues only, village development committees (VDC) dealing with overall developmental (including health) issues, community based organisations (CBO) dealing with HIV and AIDS issues, and home-based care (HBC) groups providing care for persons who are chronically ill. Membership of such groupings is open and voluntary. Most groups pursued specific common interests, and some were set up due to gender ascribed roles such as grave diggers and fishing (men) and cookery, religious and traditional dance troupes (women). However, due to heavy sensitization campaigns of gender equality, there were not many functions carried out exclusively by either males or females. Despite some gender ascribed roles there was a general consensus that everyone regardless of gender or age could participate in development and health activities. Some minority religious sects in both districts did not allow their members to seek medical care when sick.

## Discussion

The present study assessed PHC in two rural Malawian districts without CDTi experience and found that despite lack of a national policy on PHC, there is a PHC system in place which is being propelled by EHP programme. There is lack of awareness of PHC concept amongst health service providers at all levels. PHC services are implemented at all levels despite that the system has several challenges which affect the implementation of services. Some of the challenges are inadequacy of supplies, shortage of personnel, poor quality of infrastructures and unavailable transport and communication equipment.

The study has revealed that health providers and beneficiaries in the study districts to a greater extent share similar but not necessarily in same order priority health issues and health interventions as outlined in Table 4. Similarities in priority health issues and interventions were mostly observed amongst providers and communities in both study districts. However, while a similarity in priority health issues between providers and communities in Mangochi was observed this was not the case in Mzimba. The agreement on priorities among the providers in the two districts can be attributed to the adherence of the national priorities as set by the EHP. The difference by the communities in Mzimba to agree with the providers can be ascribed to the fact that the district has better socio-economic, demographic and health indicators than Mangochi (Table 1) which might have influenced their perceptions.

The study has also found that though some health implementation partners are present at district level, they are scarcely or unevenly distributed at health facility and community levels. Both the providers and beneficiaries perceived that PHC is not comprehensively delivered and need to be strengthened by more community participation and involvement. In both study districts, there are several socio-political groups which are actively involved in the delivery of PHC at community level.

Other previous studies have pointed out that Malawi has a viable and active PHC system in place, although not without problems [16-18]. Observations in the study districts revealed inadequate population coverage of health services and care. System-related barriers to access to health services exist in the form of insufficient supplies or stock-outs of essential drugs, medical supplies and inadequate human resource for health, particularly in rural areas [19]. There are also geographical barriers to accessing health care services in that the few functional health facilities are situated far apart and many people travel long distances to access services. Consequently, stakeholders perceived serious unmet gaps in the provision of basic health services in both districts.

The study found that although there is a PHC system which spans from community, through health centre up to district levels in the study districts, it lacks proper and effective coordination. The general lack of awareness of the PHC and EHP concepts among health service providers and consumers, pointed to weak policy formulation and dissemination within the system, resulting in implementation disparities. Implementation and organisation of PHC interventions at community level suffered from inadequate resources, poor collaboration between partners and limited participation of the communities benefiting from the services. It is recognised that by the adoption of the EHP by the Ministry of Health has helped to revitalise the dormant PHC strategy in Malawi.

As one way of strengthening PHC at community level, other well tested strategies such as CDI could be explored [20]. A wealth of literature shows that interventions against conditions such as malaria, diarrhoeal diseases and malnutrition can realistically be managed at community level, whereas others such as measles and HIV and AIDS require substantial medical training and experience to be properly managed [14,18,19,21-23]. It is therefore necessary to distinguish health issues suitable for community directed management from those that should remain in the hands of professional workers at health facility level. This study has documented PHC resources, capacities and supportive systems that are assets in promoting and strengthening community participation in PHC related services at community level. Based on these assets, we propose intensified dialogue with communities and public health authorities in the districts on which interventions are suitable for implementation using the CDI approach and how to do it in practice. Special emphasis in this dialogue should be paid to interventions identified as promising by informants of this study, namely:

1. Insecticide treated nets

2. Home case management for malaria

3. Management of diarrhoeal diseases

4. Treatment of schistosomiasis

5. Provision of food supplements against malnutrition.

For those interventions that we do not consider suitable for the CDI approach, some further operational research studies that would assess their feasibility for the CDI approach or shed light on ways of making them suitable for the CDI approach need to be done.

It is noteworthy that despite the many challenges faced by the health sector in Malawi, the authorities remain committed to improving accessibility to community health services. There are ongoing community-based health initiatives actively engaging communities themselves [24]. There is also a network of volunteers and community health workers who are actively involved in the delivery of various health services at community level. However increasing community participation is likely to bring specific programme benefits, as shown with CDI [13,14]. Here we show that both providers and beneficiaries in the two study districts perceived community participation as vital for the strengthening of PHC. It is desirable that the interventions are defined on the basis of existing supportive capacity and engagement of the health system, its partners, and the communities themselves to increase the sense of ownership and to minimise conflicts or duplication of efforts [25]. This study shows that perspectives of stakeholders on the specific PHC interventions to be prioritized were similar. Thus, the minimum conditions for the early involvement of all stakeholders, including community members, in the planning phase of interventions are met. Stakeholders interviewed expressed their desire for the implementation of interventions such as provision of ITN to children under the age of five and ante-natal mothers, home case management of malaria, management of diarrhoeal diseases, treatment of schistosomiasis, HIV testing and counselling (HTC), prevention of mother to child transmission (PMTCT) of HIV services and provision of food supplements to malnourished children and mothers implemented at the community level. However, considering the limitations in available resources, competencies as well as supportive systems in the target districts it is more realistic to apply the CDI strategy for the provision of ITN, home case management of malaria, management of diarrhoeal diseases and treatment of schistosomiasis than for the provision of HTC and PMTCT services, and food supplementation.

Our study has some limitations. Firstly, qualitative data were collected from only two districts. Our results therefore may not represent the whole country, though interviews with national authorities and reviews of official documents suggest that our findings could be generalised. Secondly, although independent FGD were conducted with different age groups and genders, no different perceptions of PHC priorities were found between them, suggesting that the issues raised were prevalent in the areas such that the old and the young, male or female knew about them leading them to identify the same priorities. Further, results of the individual in-depth interviews reported similar observations, which support our findings. The use of multiple data sources is a major strength of this study. There is a need for intervention studies applying both qualitative and quantitative methods to assess the processes and effects of specific CDI interventions and for those interventions where CDI strategy has never been applied there is a need for further research to assess their feasibility or to shed light on ways of making them suitable for the CDI approach.

## Conclusion

Our findings indicate that while there is in existence a PHC structure in the two target districts, the implementation is sub-optimal. However, there is a political will on the part of the Government of Malawi to improve PHC implementation at community level and to ensure larger community participation. When coupled to the expressed willingness by the community to participate and contribute towards the planning and implementation of PHC this could provide a fertile environment for efforts to intensify community participation. This study has identified several PHC related interventions that may realistically be implementable at community level using a CDI approach. The next logical step would be to evaluate these interventions in practice with emphasis on their added value in strengthening PHC interventions at community level, especially for its capacity to increase workers motivation, quality of care and programme sustainability.

## Competing interests

The authors declare that they have no competing interests.

## Authors’ contributions

PM and PB conceived the project. PM, PB, ASM, HB, GBM, EN, SJ and CM designed the study, designed the tools, selection and preparation of study areas. HB and GBM trained and supervised data collectors. PM, PB, ASM, ADS, HB and GBM analysed and interpreted the data. All authors participated in writing and approved the final manuscript.

## Pre-publication history

The pre-publication history for this paper can be accessed here:

http://www.biomedcentral.com/1472-6963/12/328/prepub
